# Reducing Ageism in a Lifespan Development Course Using Service Learning: Does an Aging Focus Matter?

**DOI:** 10.1093/geroni/igaa057.024

**Published:** 2020-12-16

**Authors:** Allyson Graf, Callie Bolling

**Affiliations:** Northern Kentucky University, Highland Heights, Kentucky, United States

## Abstract

A rapidly growing older adult population underlies the importance of reducing ageism. Research shows that college students typically hold negative views of older adults. While education and demonstrations within aging-focused courses contribute to reducing ageist beliefs, attitudes, and behavior toward older adults, contact with older adults amplifies these effects. This study investigated whether integrating contact with older adults into a lifespan development course was effective in decreasing college students’ ageism. The sample (N = 104; Mage = 19.94, SD = 3.27) were enrolled in the psychology course, largely as a major requirement for social science majors (51.9%) and health science majors (38.5%). As part of the course curriculum, a portion of the students (n = 57) interacted with older adults to fulfill a service-learning requirement; a control group (n = 47) were not given this option. Knowledge (Facts on Aging), behavior (Relating to Old People Evaluation; aging-related career intentions), and attitudes (Anxiety about Aging and Ambivalent Ageism) were assessed at the beginning and end of the semester. Quantity and quality of contact with older adults was also measured at baseline as a covariate. In a series of ANCOVA analyses, students with aging-related experiences across the term did not differ significantly on any measure compared to those without these experiences, controlling for experience with older adults and baseline assessments. The implications of this finding in the context of research may signal that focus of the course content may be an important moderator of the effectiveness of service-learning experiences with older adults.

